# Psychogenic Polydipsia in a Patient With a Clinical Triad

**DOI:** 10.7759/cureus.26651

**Published:** 2022-07-07

**Authors:** Joshua T Dodge, Ariel Kidron, Benjamin W Cooper, Adam Shepard

**Affiliations:** 1 Psychiatry, Dr. Kiran C. Patel College of Allopathic Medicine, Fort Lauderdale, USA; 2 Emergency Medicine, Dr. Kiran C. Patel College of Osteopathic Medicine, Fort Lauderdale, USA; 3 Internal Medicine, Aventura Hospital and Medical Center, Aventura, USA

**Keywords:** hypo-osmolality, acute hyponatremia, syndrome of inappropriate antidiuresis, schizophrenia and other psychotic disorders, primary polydipsia

## Abstract

Psychogenic Polydipsia (PP) is a condition involving excessive fluid intake causing hyponatremia. While the mechanism is unknown, treating arginine vasopressin (AVP) dysregulation with the class of drugs, vaptans, during acute psychotic episodes has been an effective treatment. These patients may present with a triad of acute psychosis, polydipsia, and electrolyte imbalances suggesting a syndrome of inappropriate antidiuretic hormone. Our patient is a 57-year-old female with a past medical history of schizophrenia who presented with seizures due to severe hyponatremia in the context of excessive water consumption and mild delusions regarding her sister. Her episodes of neural dysfunction started after she stopped taking her antipsychotic medications, making a drug-induced syndrome of inappropriate antidiuretic hormone secretion (SIADH) less likely. However, she had a normal urine osmolality raising suspicion of antidiuretic hormone involvement. The mechanism of hyponatremia in the context of polydipsic schizophrenia is not well established. Some evidence suggests that brain changes may cause AVP dysregulation, which can be exacerbated by acute psychotic episodes. Our case report describes a clinical scenario with the clinical triad of acute psychosis, polydipsia, and electrolyte imbalances suggestive of this mechanism.

## Introduction

Psychogenic polydipsia (PP) is a disorder where dramatically increased free water consumption leads to hyponatremia. The disease progresses in three main stages: simple polydipsia with polyuria, hyponatremia from polydipsia, and physical complications from water intoxication, such as muscle cramping, confusion, and double vision [1]. While the mechanism is poorly understood, PP has an increased prevalence in patients with psychotic illnesses with 18% of chronic schizophrenia patients afflicted [2]. Other mental disorders such as anxiety, intellectual disability, and personality disorders are also associated with PP [3-5]. As many as 6% to 20% of patients with a psychiatric diagnosis may be affected by psychogenic polydipsia [2-3]. Diagnosing PP can be difficult because a competing diagnosis, syndrome of inappropriate antidiuretic hormone secretion (SIADH), is a common adverse event of many psychiatric medications. Thereby making it unclear if the psychiatric patient’s hyponatremia is from PP or drug-induced SIADH. The literature contains a few case reports describing a triad of acute psychosis, polydipsia, and electrolyte imbalance consistent with SIADH [6] (see Appendices). Studies have investigated the relationship between acute psychosis and antidiuretic hormone (ADH) regulation as a possible pathophysiology [7]. We present a case report of a patient with concomitant intellectual disability and chronic schizophrenia who presented with the relevant clinical triad.

## Case presentation

A 57-year-old female with a past medical history of schizoaffective disorder, intellectual disability, hypertension, hyperlipidemia, and diabetes mellitus presented to the hospital after undergoing a witnessed, one-minute generalized tonic-clonic seizure with tongue biting and urinary incontinence. The seizure was unprovoked and occurred while she was sitting on her couch. She did not strike her head. After the seizure, she was unresponsive to her sister. The patient lives at home with her sister who acts as her primary caregiver. Upon arrival at the ER, her serum sodium was 106mmol/L and she was transferred to the ICU without intubation. There was no evidence of acute infarct, intracranial hemorrhage, midline shift, mass effect, extra-axial collection, acute fracture, or acute trauma-related deformity of the cervical spine. Her serum sodium concentration was brought up to 118mmol/L within the first seven hours of her hospitalization which stabilized her condition. Her serum osmolality, urine osmolality, and urine sodium concentration were 228mOsm/Kg, 194mOsm/Kg, and 34mmol/L, respectively, suggesting possible SIADH.

The patient first began to develop neural symptoms when she was unable to refill her psychiatric medications. She went without medications for seven days before she arrived in the ER. She began to develop ataxia and recurrent falls in the context of excessive water consumption, upwards of 3L of water per day. Further, this was her fifth hospital visit due to recurrent falls. Her home medications include risperidone, trazodone, citalopram, valproic acid, venlafaxine, benztropine, and paliperidone palmitate (last given four months prior). However, her sister reports poor compliance with these medications. The patient reported polyuria and appears withdrawn and irritable during water restriction. Her mood improved whenever she received water. She stated that her sister is a “demon” and showed little understanding or concern for her hyponatremia. She denied loss of interest, guilty feelings, low energy, low appetite, suicidal/homicidal ideation, manic episodes, auditory or visual hallucinations, access to guns, alcohol use, history of tobacco use, or illicit drug use. The differential included hyponatremia from SIADH or PP. Given that the urine osmolality was not maximally dilute, SIADH could not be ruled out. However, since the onset of symptoms occurred after she stopped taking her medications and she had increased water consumption, drug-induced SIADH was less likely. The patient was counseled about the importance of maintaining medication compliance and restricting water intake. Her sister was also informed to help limit the patient's excessive water consumption.

## Discussion

Psychogenic polydipsia can be a life-threatening disease and has a strong prevalence with chronic schizophrenia. Specific treatments beyond fluid restriction are limited as little is known about the pathophysiology. One possible mechanism is related to a reset in the osmoregulation of the hypothalamic-pituitary axis (HPA). Sensitive studies showing indirect evidence of arginine vasopressin (AVP) dysregulation and evidence of increased AVP during psychotic episodes first supported such a hypothesis [7]. One such study reports vasopressin antagonists may be an efficacious acute treatment. This is further supported by the rapid improvement of hyponatremia in polydipsic schizophrenic patients given a vasopressin V2-receptor antagonist [8]. Studies investigating polydipsic, hyponatremic schizophrenic patients and polydipsic, normonatremic schizophrenic patients demonstrated that acclimation to a stressful in-patient environment helped normalize the AVP dysregulation, suggesting stress may play a role in the AVP dysregulation [9]. The relationship between acute psychotic episodes and AVP dysregulation was further investigated by monitoring AVP levels during an induced psychotic episode. The AVP level was transiently increased during the psychotic episode [9]. Together, these suggest that AVP dysregulation plays a role in PP in schizophrenia and that acutely stressful and psychotic states may exacerbate such conditions. Brain studies investigating these patients show decreased anterior hippocampus and left insula volume and propose a possible mechanism. The anterior hippocampus is responsible for constraining the HPA axis and AVP responses to stress. The anterior hippocampus is also suspected to play a role in schizophrenia [9]. These studies suggest a possible mechanism for potentially life-threatening hyponatremia that is produced by increased ADH secretion and polydipsia. One would expect a patient with PP and normal AVP regulation to have urine osmolality at a maximally dilute concentration (100 mOsm/Kg). However, our patient had a urine osmolality almost twice as concentrated (194 mOsm/Kg), making the distinction between SIADH and PP more difficult. This situation has been reported in other case reports and integrates well with the possible pathophysiology described in the literature [6,9,10]. This mechanism gives light into a perplexing clinical triad: acute psychosis, polydipsia, and electrolyte imbalances suggesting SIADH.

## Conclusions

In summary, our patient had a diagnosis of schizoaffective disorder, was non-compliant with medication and subsequently became polydipsic prior to hospitalization. Our case report redemonstrates the clinical triad of acute psychosis, polydipsia, and electrolyte imbalance that suggests this possible mechanism. Further investigation into this pathophysiology can better the understanding and improve treatment modalities for this disease.

## Appendices

**Table 1:** Patients hospitalized for polydipsic water intoxication without maximally dilute urine as found in the literature *Measurement taken on third day of admission, after serum sodium was increased to 122 mEq/L

Age	Gender	Psychiatric Diagnosis	Serum Sodium (mEq/L)	Serum Osmolality (mOsm/L)	Urine Sodium (mEq/L)	Urine Osmolality (mOsm/L)	Citation
57	Female	Schizoaffective disorder	106	228	34	194	Our patient
52	Female	Prior manic psychosis, presented with current psychosis	100	248*	37	397*	Raskind et al.,1975 [6]
53	Female	Delusion of persecution with agitation 3 weeks prior without hyponatremia	106	228	52	263	Raskind et al., 1975 [6]
63	Female	Schizophrenia	124	250	62	486	Raskind et al., 1975 [6]
52	Male	Schizophrenia	125	266	4.5	620	Fowler et al., 1977 [10]
50	Male	Schizophrenia	112	253	24	258	Fowler et al., 1977 [10])
48	Male	Schizophrenia	105	215	--	260	Fowler et al., 1977 [10]
